# Supplementary material for: Acid–Base Equilibrium and Self-Association in Relation to High Antitumor Activity of Selected Unsymmetrical Bisacridines Established by Extensive Chemometric Analysis
Source: Molecules. 2022 Jun 21;27(13):3995. doi: 10.3390/molecules27133995 (PMC9268451; doi:10.3390/molecules27133995)
Supplement: Supplementary file 1 [file molecules-27-03995-s001.zip › molecules-1779890-supplementary.pdf]

Supplementary Materials

Extensive chemometric analysis exploring the acid-base equilibrium and self-association of unsymmetrical bisacridines reveals characteristics possibly relevant for their anticancer activity

Michał Kosno[†], Tomasz Laskowski[†,*], Joanna E. Frackowiak[†], Agnieszka Potęga[†], Agnieszka Kurdyn[†], Witold Andrałojć[‡], Julia Borzyszkowska-Bukowska[†], Katarzyna Szwarc-Karabyka[§], Zofia Mazerska[†,*]

[†] Department of Pharmaceutical Technology and Biochemistry and BioMedTech Centre, Faculty of Chemistry, Gdańsk University of Technology, Narutowicza Str. 11/12, 80-233 Gdańsk, Poland

[‡] Institute of Bioorganic Chemistry, Polish Academy of Sciences, Noskowskiego Str. 12/14, 61-704 Poznań, Poland

[§] Nuclear Magnetic Resonance Laboratory, Narutowicza Str. 11/12, 80-233 Gdańsk, Poland

* Correspondence to: tomasz.laskowski@pg.edu.pl (T.L.), zofia.mazerska@pg.edu.pl (Z.M.)

1 Chemometric analysis

1.1 Chemometric analysis – general procedures.

All absorbance values were expressed as molar extinction coefficients. Values were organised in matrix W containing *n* rows (wavelengths) and *m* columns (spectra obtained in different pH conditions). Firstly, data was centred according to the equation provided below (S1) resulting spectra are presented in Figures S1 and S2:

$$W_{ij} = S_{ij} - \mu_j \qquad (S1)$$

W_{ij} – centered molar extinction coefficient of i-wavelength and j-spectra,

S_{ij} – molar extinction coefficient of i-wavelength and j-spectra,

μ_j – the mean value of the molar extinction coefficient of the j–spectra.

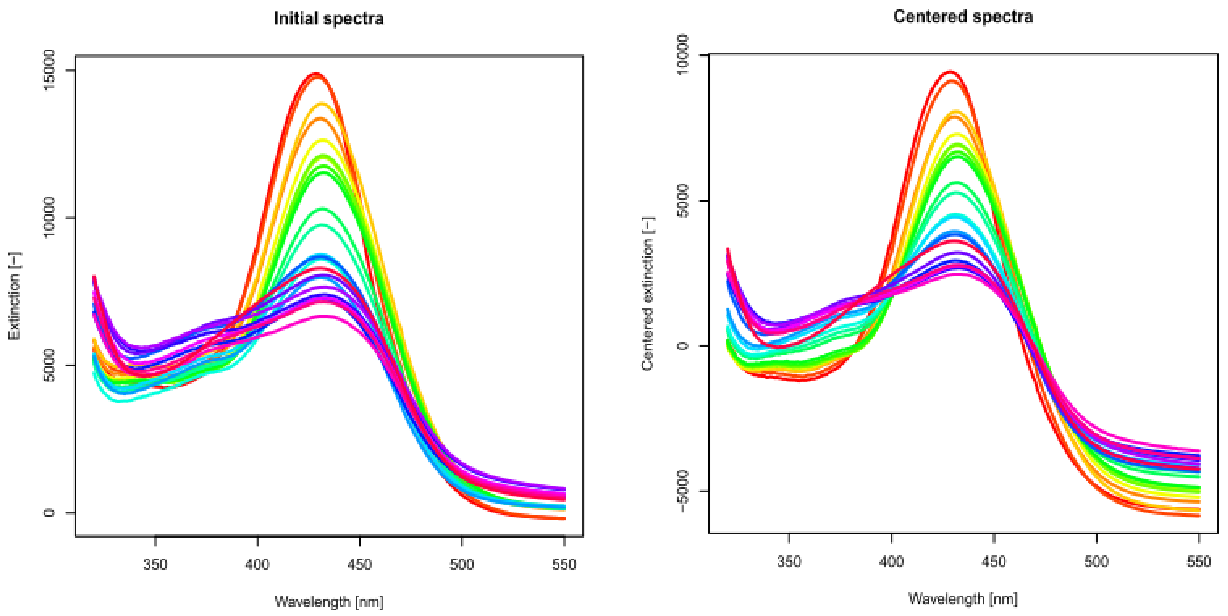

Figure S1. Spectra for analysis of C-2045 protonation forms. Initial spectra (left), centered spectra (right).

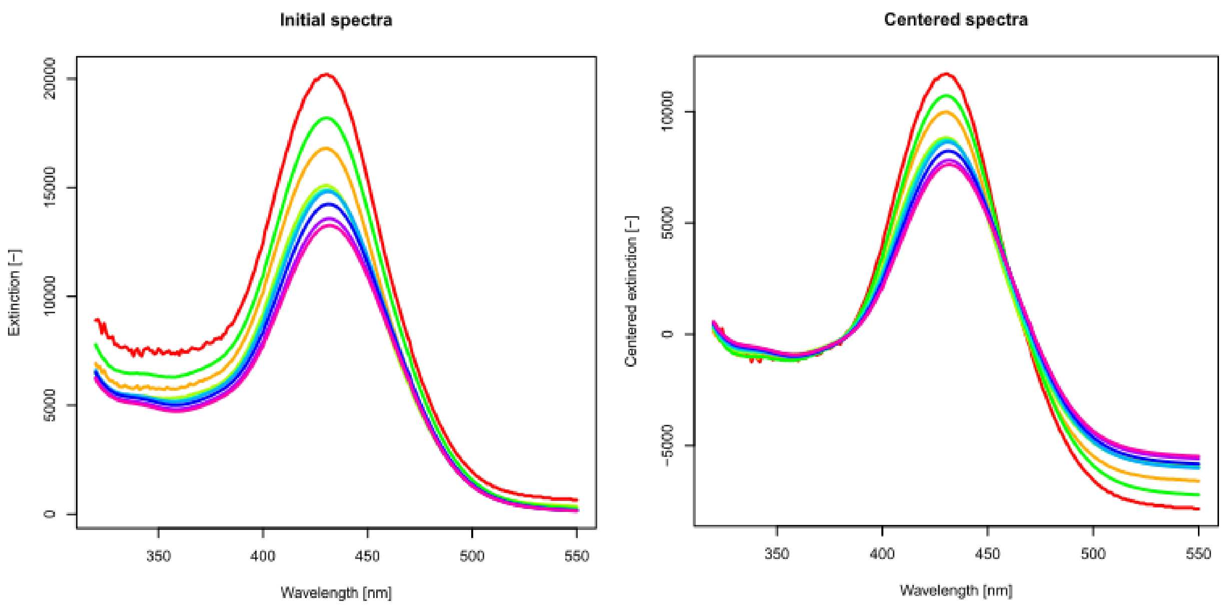

Figure S2. Initial spectra for aggregation study of C-2045 (left), centered spectra (right).

1.1.1. Singular value decomposition of the matrix.

The next step was principal component analysis of the *W* matrix. *W* matrix was converted into a covariance matrix (S1) and decomposed.

$$cov(x,y) = \frac{1}{n-1}\sum_{i=1}^{n}(x_i - \bar{x})(y_i - \bar{y}) \quad \text{(S2)}$$

x,*y* – variables,

n – number of wavelengths.

Covariance matrix W was decomposed into singular values (according to the SVD algorithm), which allowed to determine eigenvalues and eigenvectors (U).

Contributions of principal components were calculated using the matrix equation provided below (S3).

$$U = [V^T V]^{-1} V^T W \quad \text{(S3)}$$

Conversely, the coordinates of the observations on the principal components are calculated by:

$$V = WU[UU^T]^{-1} \quad \text{S4)(}$$

V – coordinates of the observations on the principal components,

W – centered spectrum,

U – contributions of principal components.

Matrices U and V were used to calculate components of the spectra (Figures S3 and S4). If a spectra matrix component is denoted by C, then it can be calculated as:

$$C = VU^T \qquad (\text{S5})$$

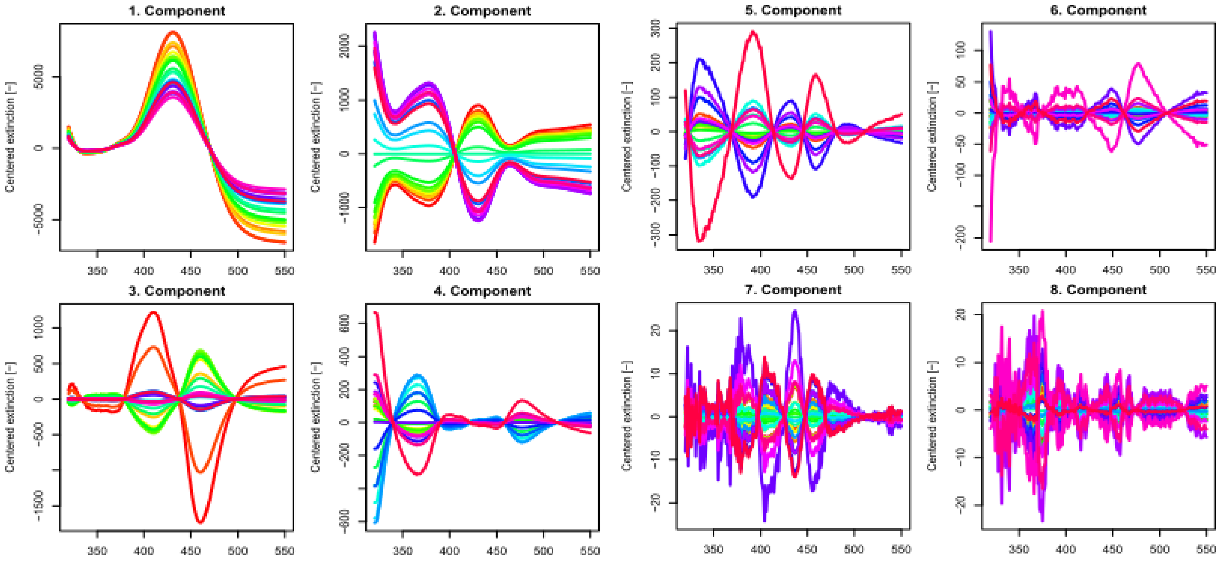

Figure S3. Spectra of C-2045 components (pK_a determination).

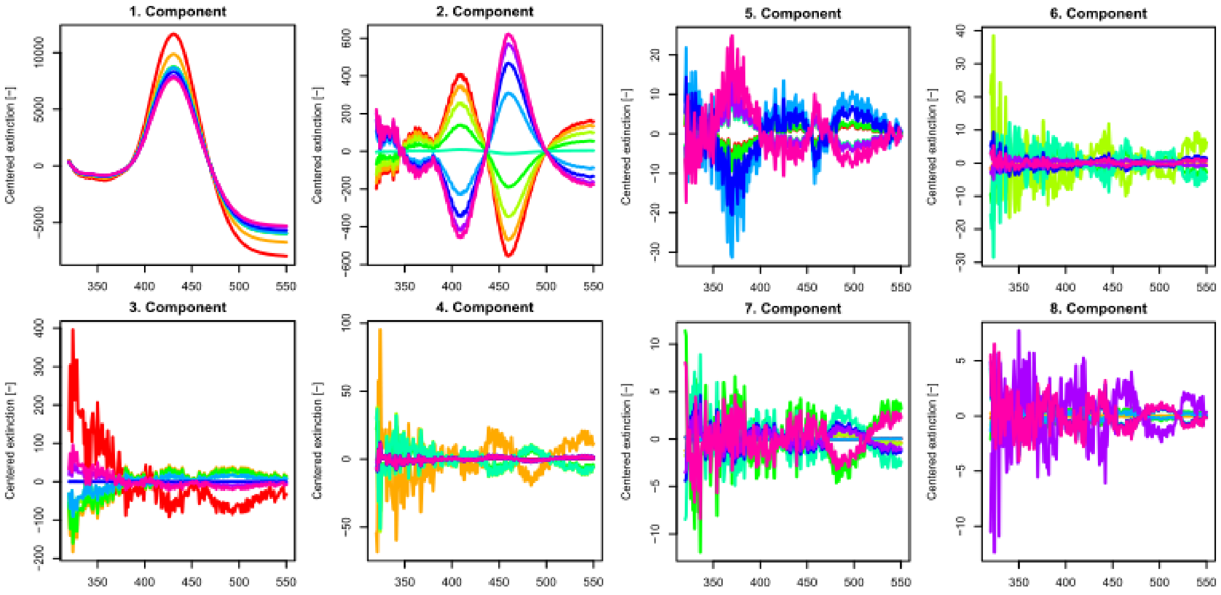

Figure S4. Spectra of C-2045 components (aggregation study).

Reconstructed spectra (D) were determined based on C, and residual spectra (R) were then calculated by:

$$R = W - D \qquad (\text{S6})$$

The spectra are placed in a vector space, which is spanned by the principal components. These vectors are placed into a hyperplane with the number of dimensions equal to the number of pure spectral forms. This means that the first step in further analysis should be determining the number of spectral forms. This can be achieved by analysing the eigenvalues and spectra of the components. It is assumed that the rank of residual spectra at which the systematic changes of these spectra disappear defines the number of significant principal components (spectral forms). Subsequently, the points corresponding to the pure spectral forms need to be identified over the principal component space.

1.1.2. Calculation of the molar fractions and individual spectrum matrix.

Calculation of the *X* matrix allows to solve equation (S7) which results in *B* matrix.

$$W = BX \qquad (S7)$$

W – initial data matrix,

B – individual spectra matrix,

X – estimation of the molar fraction matrix.

Values of the *U* matrix were optimised using the penalty function. Optimised values obtain a penalty for violation of the constrains. This means that it is required to add a value until a minimum of the optimised function (FC) is achieved.

$$penalty = \sum\sum x_{ij}^2 \qquad (S8)$$

$$reward = \sum_i var(x_{ij}) \qquad (S9)$$

$$FC = penalty - reward \qquad (S10)$$

Based on the selected number of significant eigenvectors, n, principal component analysis was performed once again to extract the (n-1)-dimensional hyperplane from the n-dimensional space. Points from the X matrix were placed into a four-dimensional tetrahedron analogue. Corners of the given entities were found manually and then optimised using the simplex method. Molar fractions were obtained based on contributions of the principal components and were calculated using coefficients of the plane Equations (S11) – (S12). Appropriate planes were determined using the linear regression method.

$$\sum_i d_i = h \qquad i \in \{R\} \; (S11)$$

$$u_i = \frac{d_i}{h} \qquad (S12)$$

h – height of a five-dimensional simplex,

d_i – distance from u_i to the opposite plane of the simplex,

u_i – molar fractions.

An example of a four-dimensional simplex is shown in Fig. S5. In this case, the quaternary plot for C-2041 is shown because it is not possible to accurately represent a projection of a higher dimensional simplex on the plane.

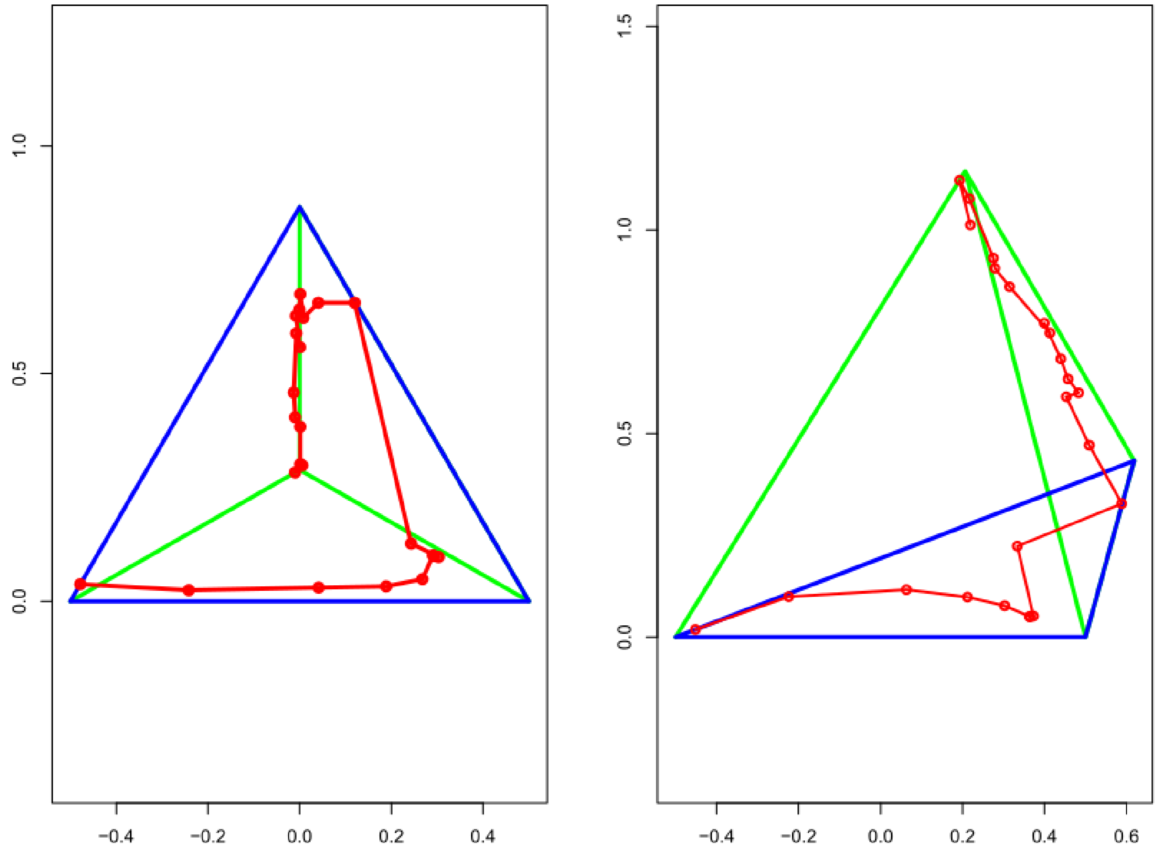

Figure S5. Quaternary plot for C-2041 in orthogonal projection (left) and perspective projection (right).

Dissociation constants (pK_a) were determined based on the molar fractions using an extended version of the Henderson-Hasselbalch Equations (S13 – S15).

1.1.3. Fitting to the theoretical model.

a) pK_a determination

Based on previously determined pKa values, theoretical molar fractions were calculated and compared to those experimentally determined.

$$\gamma = \frac{\prod_{i=1}^{4} K_i}{\prod_{i=1}^{4} H^i} \qquad (S13)$$

$$u_1 = \frac{1}{1+\gamma} \qquad (\text{S14})$$

$$u_n = u_1 \cdot \gamma \qquad n = \{1,4\} \qquad (\text{S15})$$

K – Dissociation constant,

u_n – Molar fractions.

In the next step, pKa values were optimised using the simplex optimisation algorithm and then were validated using *leave-one-out* cross-validation.

All numerical optimisations were performed using the Nelder-Mead algorithm, which is based on comparing the objective functions in the m+1 corners of the simplex and moving it towards the extremum. A simplex is an n-dimensional object, which is the smallest convex set containing points p_0, p_1,…, p_n, which are the corners of the simplex.

The maximum number of iterations was 10 000.

b) Self-association study

Based on determined molar fractions, theoretical molar fractions were calculated using an appropriate aggregation model (dimerization or unlimited aggregation) and fitted to those experimentally determined. All calculations were performed in the R environment (version 4.0.2).

2 NMR assignments for UAs

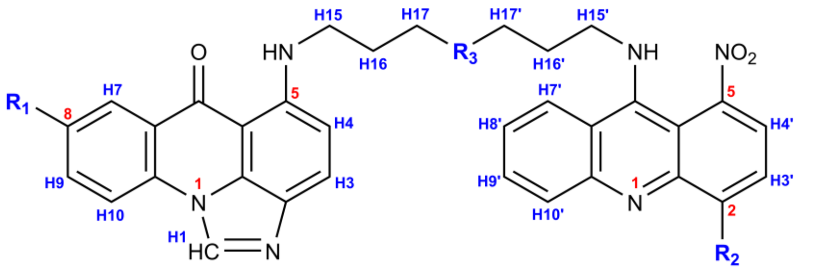

Figure S6. The general structure of UAs with proton codenames, used in NMR assignments.

Table S1. Chemical shifts (δ) of resonance signals for non-exchangeable protons in UAs, recorded at pH = 4.5, 25°C in D_2O. Multiplet codenames: s – singlet, d – doublet, t – triplet, m – unresolved multiplet. All chemical shifts were referenced to residual water signal at δ = 4.780 ppm. Protons were referenced as shown in Figure S6.

	^{1}H chemical shift [ppm] (observed multiplet)			
**Proton** *(amount)*	**C-2028**	**C-2041**	**C-2045**	**C-2053**
**H1** *(1H)*	8.093 (s)	8.267 (s)	8.096 (s)	8.069 (s)
**H3** *(1H)*	7.139 (d)	7.435 (d)	7.364 (d)	7.159 (d)
**H4** *(1H)*	6.473 (d)	6.365 (d)	6.543 (d)	6.450 (d)
**H7** *(1H)*	7.299 (d)	7.523 (d)	6.181 (s)	7.211 (d)
**H8** *(1H)*	7.194 (t)	7.128 (t)	-	7.141 (t)
**H9** *(1H)*	7.669 (t)	7.517 (t)	6.921 (d)	7.654 (t)
**H10** *(1H)*	7.457 (d)	7.375 (d)	7.174 (d)	7.414 (d)
**H15** *(2H)*	3.487 (m)	3.291 (m)	3.479 (m)	3.472 (m)
**H16** *(2H)*	2.132 (m)	2.119 (m)	2.173 (m)	2.118 (m)
**H17** *(2H)*	3.308 (m)	3.355 (m)	3.241 (m)	3.291 (m)
**H2'** *(1H)*	7.674 (d)	7.605 (d)	-	-
**Me2'** *(3H)*	-	-	2.488 (s)	2.566 (s)
**H3'** *(1H)*	7.768 (t)	7.655 (t)	7.596 (d)	7.596 (d)
**H4'** *(1H)*	7.872 (d)	7.789 (d)	7.845 (d)	7.772 (d)
**H7'** *(1H)*	7.206 (d)	7.798 (d)	7.011 (d)	7.616 (d)
**H8'** *(1H)*	7.020 (t)	7.301 (t)	7.494 (t)	7.563 (t)
**H9'** *(1H)*	7.583 (t)	7.704 (t)	7.585 (t)	7.053 (t)
**H10'** *(1H)*	7.345 (d)	7.407 (d)	6.915 (d)	7.159 (d)
**H15'** *(2H)*	3.566 (m)	3.598 (m)	3.504 (m)	3.551 (m)
**H16'** *(2H)*	2.199 (m)	2.060 (m)	1.934 (m)	2.187 (m)
**H17'** *(2H)*	3.482 (m)	2.952 (m)	3.238 (m)	3.472 (m)
**NMe** *(3H)*	3.033 (s)	-	2.999 (s)	3.024 (s)
**Lin1** *(4H)*	-	3.501 (m)	-	-
**Lin2** *(4H)*	-	3.294 (m)	-	-

3 Selected regions of 2D NMR spectra for UAs

DQF-COSY, TOCSY and ROESY experiments were conducted for resonance assignment to respective protons. We present selected regions spectra below as an example.

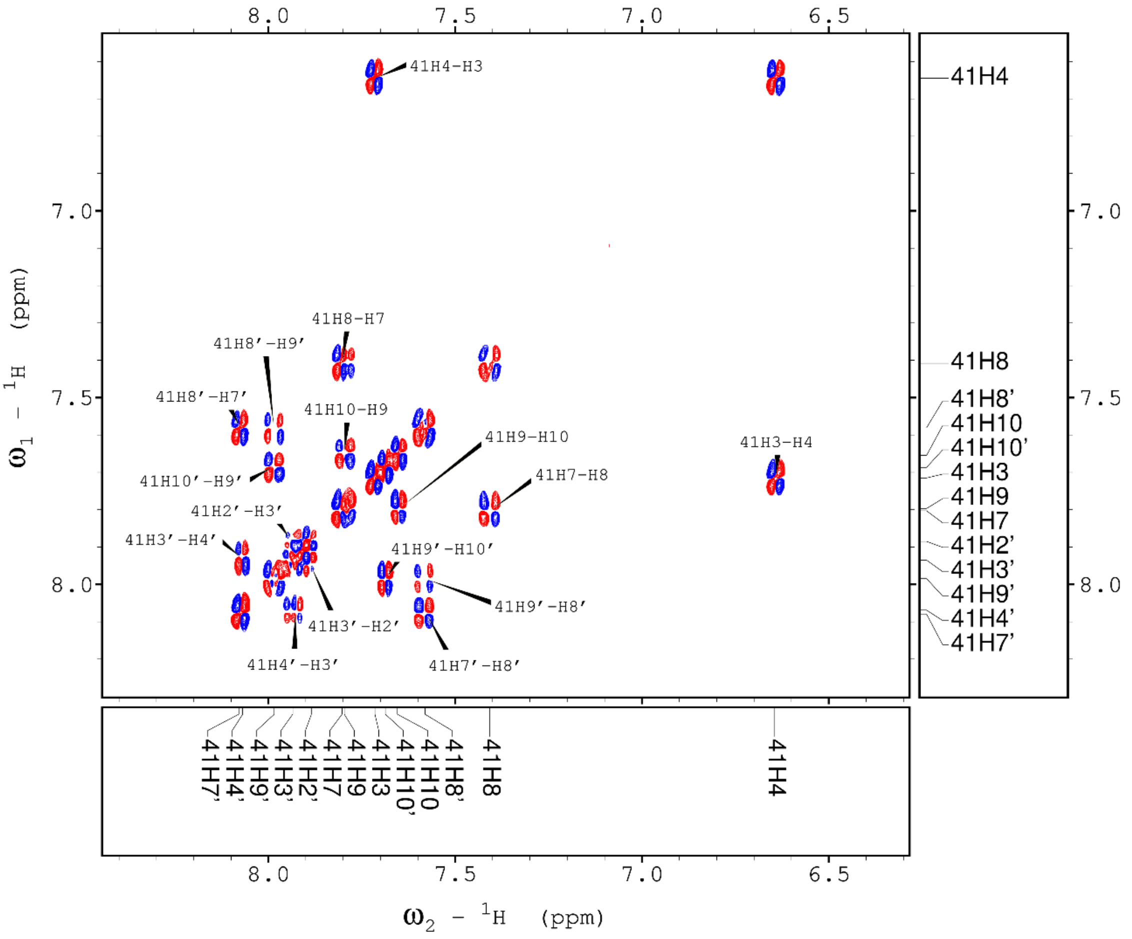

Figure S7. Aromatic region of the DQF-COSY spectrum for C-2041.

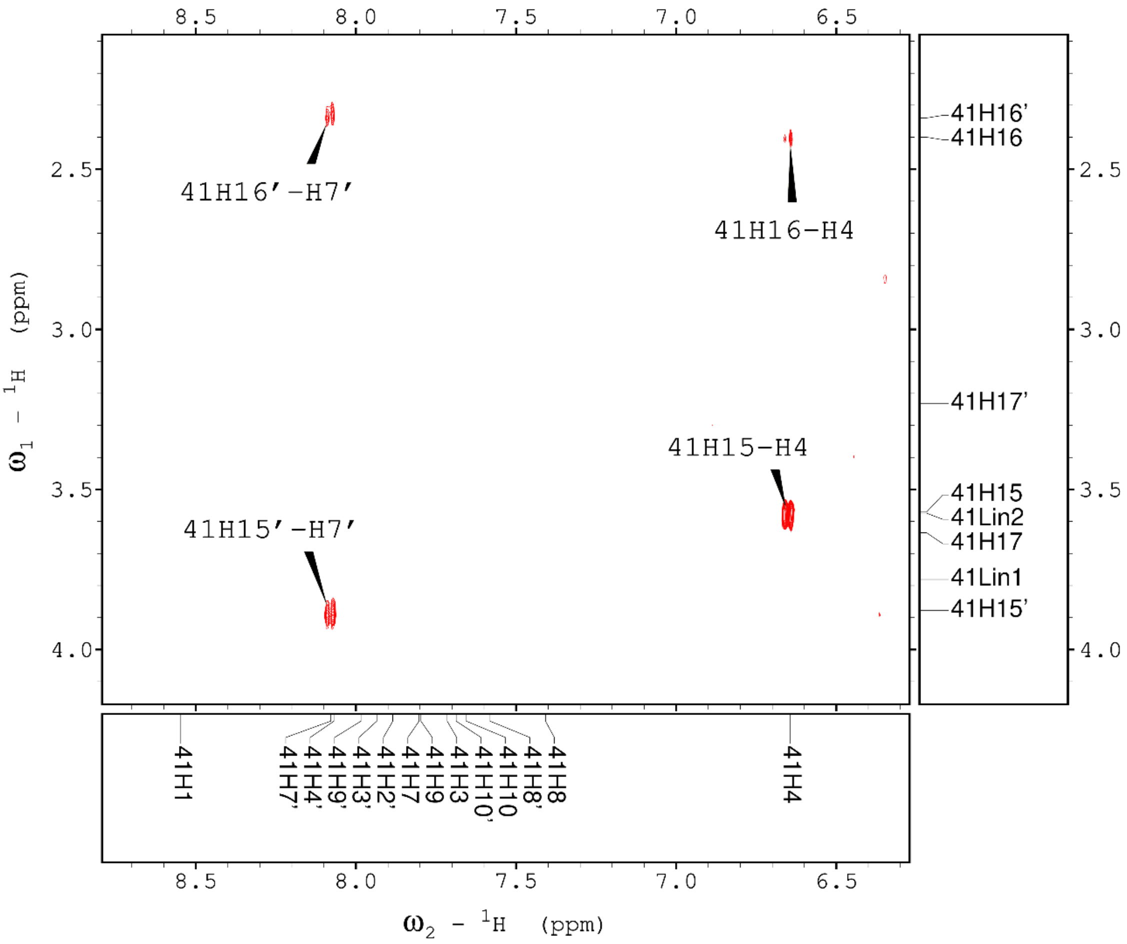

Figure S8. Fragment of the ROESY spectrum for C-2041, depicting dipolar couplings between the aromatic protons and the linker sidechain.

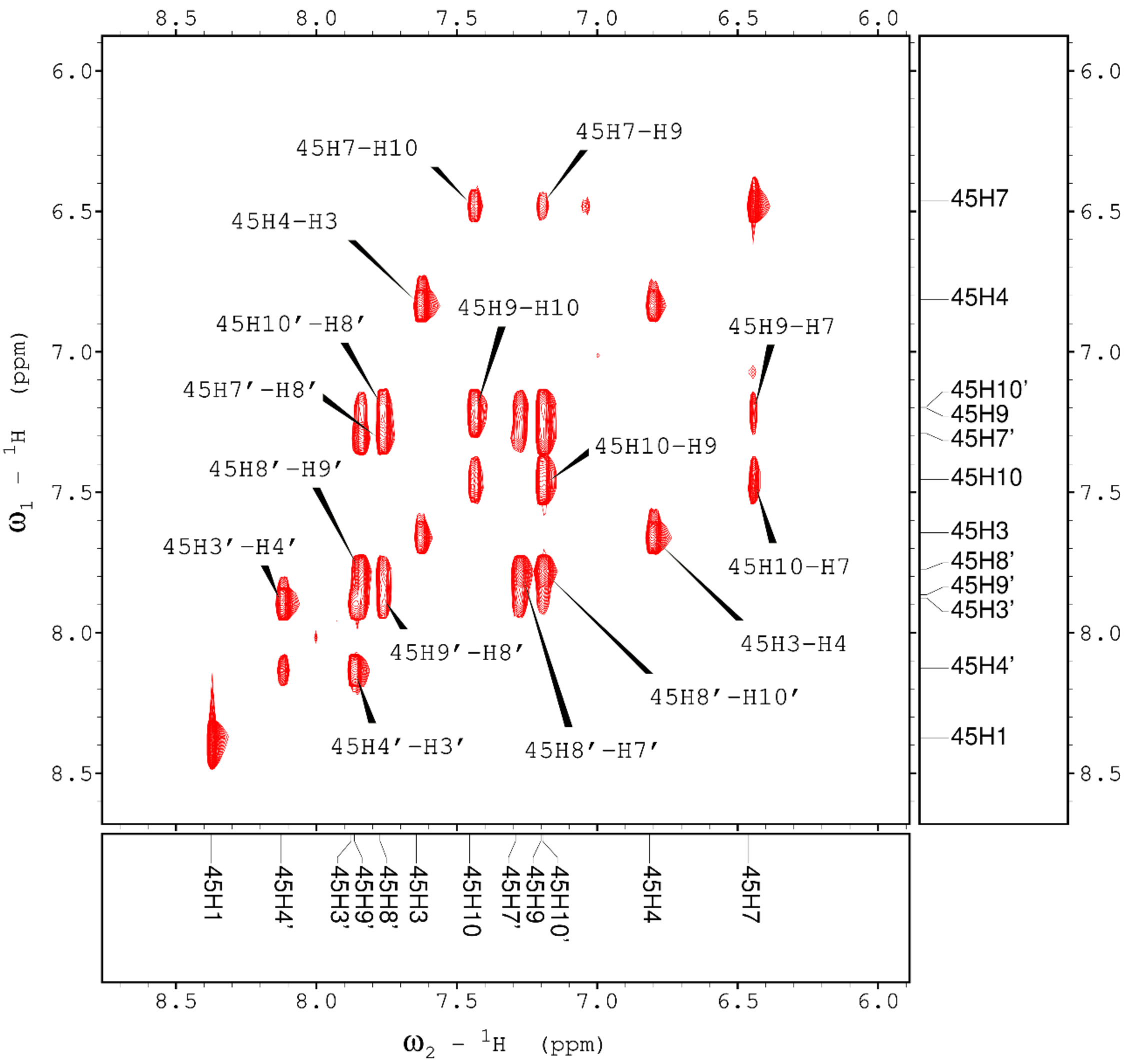

Figure S9. Aromatic region of the TOCSY spectrum for C-2045.

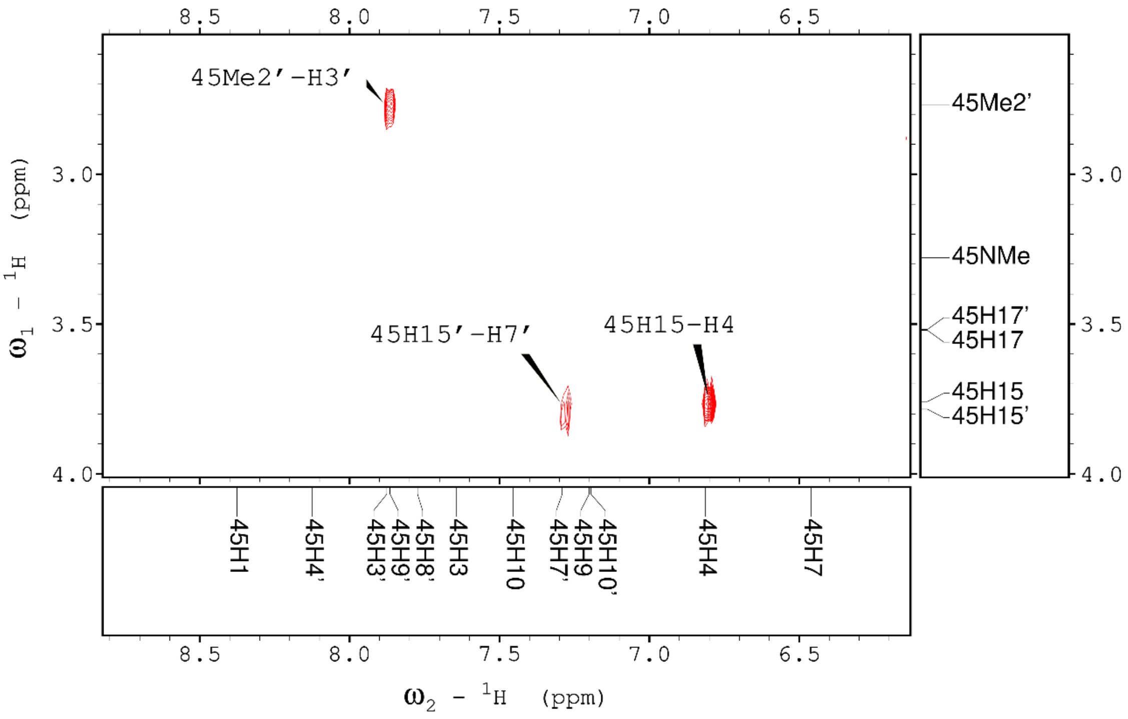

Figure S10. Fragment of the ROESY spectrum for C-2045, depicting dipolar couplings between the aromatic protons and the aminoalkyl linker. Also, diagnostic Me2’-H3’ ROE could be observed.
